# HERA: Bioinspired Separation of Local Fault Response and Language-Model Scheduling in Simulated Quadruped Control

**DOI:** 10.3390/biomimetics11090669

**Published:** 2026-09-17

**Authors:** Dongyeon Kim, Hyunjun Jung

**Affiliations:** Department of Software Science & Engineering, Kunsan National University, Gunsan 54150, Republic of Korea; 2101371@kunsan.ac.kr

**Keywords:** bioinspired control, local fault response, language-model scheduling, simulation, reproducible evaluation

## Abstract

Language-model inference can delay emergency supervision, but cancellation alone cannot provide an immediate physical response. HERA (Hierarchical Event-driven Reflex Architecture) separates a measurement-driven deterministic local action from language-service scheduling, with biological inspiration limited to temporal separation and local feedback. A fixed 1472-trial campaign evaluated canonical-command scheduling, simulated actuator faults and live integration. Across three workloads, the mean validated-command latency was 0.486–0.512 s with preemption, 1.476–11.101 s with single-lane FIFO and 0.543–0.575 s with two-slot serving. The original detector identified all 544 complete faults but missed all 160 half-strength faults. A separately frozen 560-trial follow-up evaluated a sustained effort-discrepancy warning without changing control. On the same 160 new half-strength trajectories, the original detector again missed every fault, whereas the warning detected all faults with mean delays of 161.0 and 155.5 ms of simulated time in clean and noisy conditions. No false onset occurred in 2400 simulated robot-seconds of new healthy exposure. This addresses conditional detection sensitivity, not improved physical recovery. In the original campaign, local support reduced the mean upright deficit but worsened some paired cases and reduced terminal speed; every physical trial met the fixed-horizon safety criterion. All 96 normal integrated supervisory commands completed 50-step holds and returned to local fallback, and local action persisted under injected transport failure. Because supervision reaffirmed the existing support array, these results establish neither additional LLM physical benefit nor general diagnosis or hardware safety.

## 1. Introduction

An inference service and a robot control loop operate on different timescales. When a language request occupies a limited-capacity backend, a new request may queue behind it. A cancellation policy can change request delivery, but a robot should not need to wait for that operation to apply a predefined local response. This distinction motivates evaluating scheduling, physical response, and integration as separate claims.

HERA combines a preset controller, a measurement-driven detector, a deterministic local support command, and a supervisory language-service interface. This study compares cancellation followed by same-lane submission with a single-lane first-in-first-out policy and an actual two-slot serving configuration. The local response is deliberately independent of those alternatives. The contribution sought is a traceable evaluation of this separation, not a new locomotor oscillator, a general diagnostic system, or a demonstration that an LLM creates better recovery controls.

The revised evaluation addresses software-buffer fault injection, successful-only latency comparisons, unverified concurrency, and conflation of valid JSON with physical recovery. The fixed main campaign establishes endpoint-specific scheduling advantages, complete-fault detection with a partial-fault sensitivity limitation and a physical-outcome ceiling, and supervisor application independent of the local action. Separate follow-up studies evaluate a degradation-warning extension on 560 new trajectories and inference-concurrency throughput in 32 paired-condition trials. These follow-ups preserve rather than replace the original outcomes. Leg-level aggregation and an audit of superseded latency batches additionally clarify the evidence and its provenance.

## 2. Related Work and Biological Scope

### 2.1. Biological Principles and Engineering Abstractions

Biological locomotor control motivates separating rapid local responses from slower contextual decisions, but does not justify a literal three-module software model of the nervous system. Central pattern generators are commonly modeled using neural or oscillator dynamics with sensory and descending modulation [1]; contemporary accounts further emphasize distributed, state-dependent coordination rather than a rigid serial hierarchy [2]. HERA’s preset trajectory executor has no oscillator dynamics, sensory entrainment, or adaptive phase mechanism. We therefore call it an *open-loop preset controller*, not a CPG.

This distinction is equally important for the detector. Cerebellar internal-model accounts concern prediction of sensory consequences and related control computations [3]. A nominal actuator-effort residual combined with proprioceptive and inertial measurements is an engineering consistency check; it is not evidence that this implementation reproduces cerebellar computation. Biomechanically grounded reflex models can generate locomotor behavior [4], whereas the local response evaluated here selects a predefined support array. Neither physiological fidelity nor equivalence to those models is claimed.

The BEAM-D methodology links biological abstraction with mechatronic design and verification [5]. We use that methodological perspective to make each inspiration, implementation choice, and validation obligation explicit in Table 1. We do not claim BEAM-D certification or present the authors’ proposed methodology as an independently conferred technical standard. In particular, cancellation of a remote inference request is a software scheduling mechanism. The cited biological literature does not establish a corresponding spinal-to-cerebellar-to-cortical cancellation pathway.

### 2.2. Layered Control, Language Supervision, and Adaptation

Concurrent layered control predates LLM robotics: Brooks described asynchronous modules in which lower-level behavior continues while higher-level capabilities are added [6]. Language-grounded skill selection [7], generated reactive policies [8], and asynchronous language-enhanced planning [9] also preclude a claim that existing LLM robot systems are uniformly synchronous. HERA’s narrower question is how a specified cancellation policy changes delivery of a known emergency command relative to a single-lane queue and a two-slot alternative on the tested backend, while a separate local controller continues operating.

Physical adaptation after damage is a different objective. For example, compensatory behavior search can restore useful behavior after robot damage [10]. Our fixed support command neither searches for a gait nor establishes recovered task performance. The live integration study tests a bounded interface between local control and language-service output; it is not a comparison against damage-adaptation algorithms.

### 2.3. Related Efficiency and Multimodal Reliability Research

SpecFlow addresses lightweight multimodal reasoning through a spectral-progressive representation [11]; it is relevant to inference-efficiency research, but does not directly test this scheduling policy. Self-Calibrated Consistency studies adversarial robustness of vision–language models [12]. Multimodal adversarial synergy and incomplete-input learning concern, respectively, coupled image/text attacks and missing multimodal information [13,14]. These works help delineate future multimodal reliability requirements. The present text-only canonical-command experiment does not evaluate their threat models, and simulated actuator noise or an injected transport failure is not an equivalent benchmark.

## 3. Materials and Methods

### 3.1. Execution Paths and Command Validity

The local control path obtains simulated actuator/proprioceptive/inertial measurements, evaluates a residual-based detector, and selects the trusted support array on the declared control tick. It does not wait for cancellation, HTTP completion, language generation, or supervisor validation. A separate event path supplies the measured event identity and monotonic timestamp to the scheduler. Full schema and canonical-contract validation precede supervisor acceptance; current generation and request identity precede application. Rejected, missing, or expired supervision leaves the local fallback in control.

**Figure 1:**
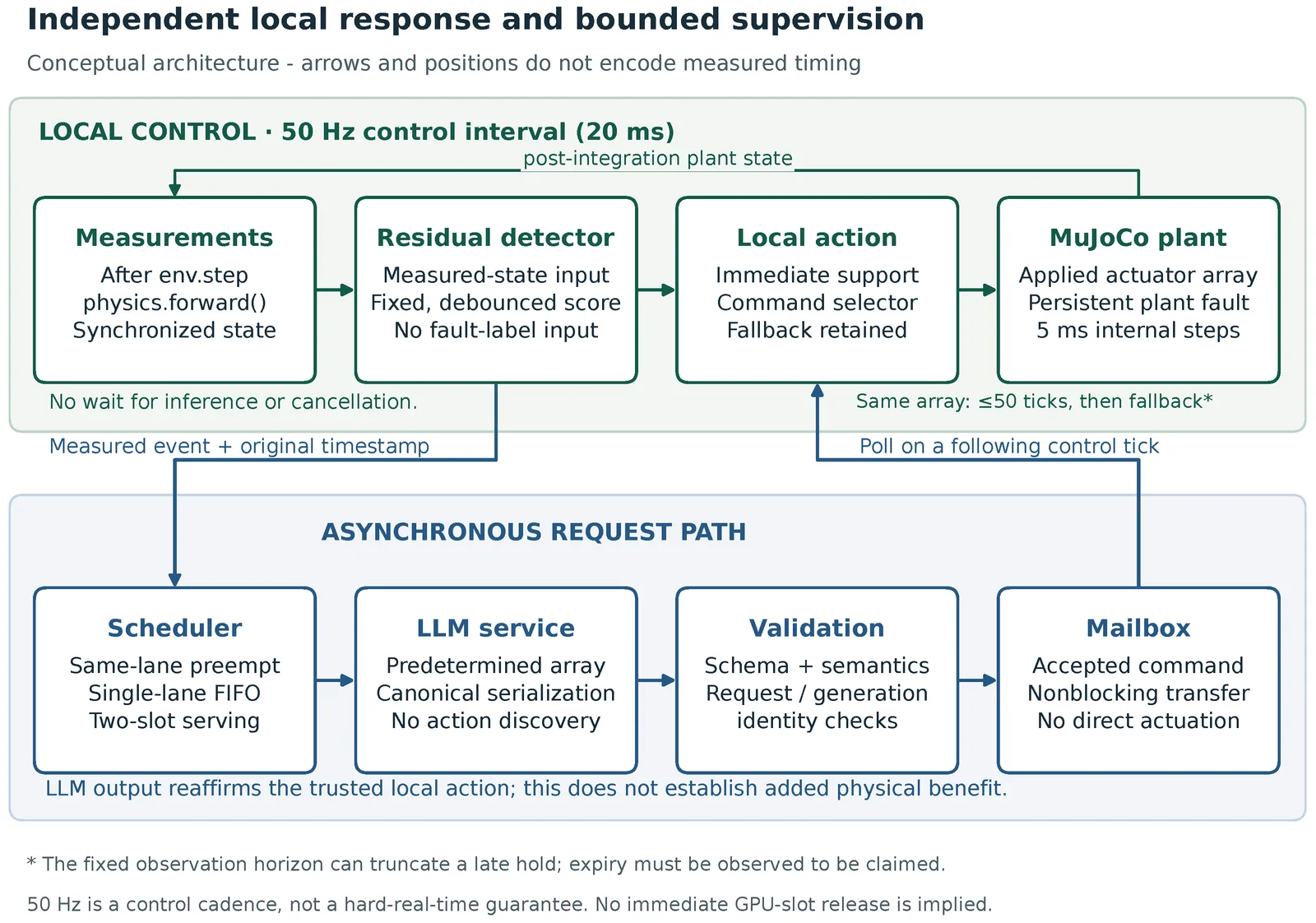
Conceptual execution paths, not measured timing. Forward-synchronized measurements drive the local response independently of request handling. An accepted canonical command is read on a following control tick and reaffirms the same support array for at most 50 control intervals; the fixed observation horizon can truncate a late hold. Arrows indicate functional connections, not instantaneous execution or GPU-slot release.

Figure 1 summarizes the separate execution paths.

The language task is intentionally constrained. Study A serializes a predefined, scenario-specific one-phase support array with duration_steps=1, a fixed skill name and stop condition, and specified one-shot fields. Study C instead requests a 50-step reaffirmation of the same support array already used locally. These contracts measure scheduling/serialization and interface application, respectively; they are not open-ended fault diagnosis or action planning. The term “trusted” identifies an allowed command, not proof that it satisfies every physical outcome criterion. Exact prompts, schema, validation rules and command arrays are part of the reproducibility materials.

### 3.2. Platform and Frozen Design

The main platform uses Windows 11 (build 26200; Microsoft Corporation, Redmond, WA, USA), CPython 3.12.0, dm_control 1.0.37, MuJoCo 3.5.0, NumPy 2.4.2 and Ollama server 0.33.2 with local llama3.2:3b in Q4_K_M quantization, identified in the archived loader log as Q4_K–Medium. The host has an Intel Core i9-13900K (Intel Corporation, Santa Clara, CA, USA; 24 cores, 32 logical processors) and an NVIDIA RTX A6000 (NVIDIA Corporation, Santa Clara, CA, USA), reporting 49,140 MiB of GPU memory with driver 595.95. The control interval is Δt=0.02 s (50 Hz), distinct from the internal MuJoCo integration step of 0.005 s. Each control interval therefore comprises four internal integration steps; step counts and duration fields refer to control intervals unless explicitly stated otherwise. Two dedicated server ports use verified parallel-one and parallel-two runners with context 8192 on the same GPU. The archived source and model-manifest SHA-256 identifiers begin 73b25c2272ad and a80c4f17acd55, respectively; the model-manifest digest is distinct from the underlying GGUF blob digest. Complete hashes, environment versions, runtime configuration and manifests are retained in campaign provenance. Documentation describes configuration facilities [15]; actual runtime records establish the settings used, not the current website.

The master seed is 20260908; the separate implementation pilot uses 20261908. Table 2 records the fixed main matrix, completed without sample expansion. Pilot observations are not pooled with the main results. Scientific failures remain outcomes; repairs or design changes after freezing require a disclosed new version/campaign.

Whole-study, workload and profile groups follow a fixed manifest order, including scheduler token caps 256, 1024 and 2048 in sequence before the physical profiles. Method and leg order are randomized within each declared batch, not across whole groups. Scenario seeds depend on the master seed, group label, block and leg; the group label includes the workload or physical profile. Exact pairing therefore applies across methods within a workload/profile, not across workloads, noise conditions or fault severities. Between-group means remain descriptive and do not isolate those factors from different seeds or temporal order.

### 3.3. Study A: Canonical-Command Scheduling

The methods are hera_preempt (cancel the mission, then use the same lane), fifo_single (wait for the mission), and reserved_slot (two client lanes with an actual parallel-two runner). The last name describes the tested configuration, not a guaranteed dedicated processor. The incumbent token budgets are 256, 1024, and 2048, analyzed separately. Each load has eight randomized complete batches of four legs and all three methods. The temperature is zero; emergency generation is capped at 512 tokens with one attempt.

The trigger is configured with a nominal 0.25 s delay after the first valid mission frame, and the mission must still be active. The achieved delay is measured from event timestamps rather than assumed to equal this setting. The absolute deadline is 30 s from the actual trigger, including cancellation, queuing, generation, and validation. Primary latency is trigger-to-first-fully validated acceptance. Events separately record enqueue/dispatch, cancellation completion, first streamed frame/content, full response, validation, and cleanup. Direct internal GPU release timing is not inferred from a client connection closure.

Every manifested failed, errored, or missing trial contributes its deadline to a descriptive penalized mean. Successful-only means, standard deviations, medians and interquartile ranges are labeled conditional. Deadline sensitivities at 1, 2, 5, 10 and 30 s reuse the observed data; they are not experiments rerun with different timeout policies. Observed token counts and mission activity/completion/cancellation are reported because equal caps do not guarantee equal realized work.

### 3.4. Study B: Physical Perturbations and Local Response

The methods are local_reflex and no_reflex. No-reflex retains the nominal trot preset; it is not zero actuation or uncontrolled motion. Initialization uses one reset, a deterministic upright yaw, and 300 stand–settle control intervals. During the 10 s measured horizon, there is no reset or restoration of the fault on an LLM response. At 3 s, an actuator fault persistently scales the selected leg’s gain and bias coefficients. The injection record stores the actual nominal and faulted parameter arrays for all three selected-leg actuators; healthy trials have no such fault snapshot. The detector uses fixed nominal parameters and measured quantities, not the ground-truth fault flag. Let $a_{i}$, $\ell_{i}$ and ${\dot{\ell }}_{i}$ be actuator activation, transmission length and transmission velocity. Its unconstrained nominal actuator-space effort is

(1) $${\tilde{f}}_{i}=g_{i0}a_{i}+b_{i0}+b_{i1}\ell_{i}+b_{i2}{\dot{\ell }}_{i}.$$

For force-limited actuators, the predicted effort ${\hat{f}}_{i}$ clips ${\tilde{f}}_{i}$ to the recorded actuator-force range; otherwise, ${\hat{f}}_{i}={\tilde{f}}_{i}$. Initialization requires fixed gains, affine biases and disabled early-activation evaluation; incompatible actuator models are rejected. For leg *L*, the force-model residual is

(2) $$F_{L}=\left\{\begin{array}{cc} {\mathrm{clip}}_{\left[0,1\right]}\;\left(\frac{\sum_{i\in L}\left|f_{i}-{\hat{f}}_{i}\right|}{\sum_{i\in L}\left|{\hat{f}}_{i}\right|}\right), & \sum_{i\in L}\left|{\hat{f}}_{i}\right|\geq 8,\\ 0, & \mathrm{otherwise}. \end{array}\right.$$

These are MuJoCo actuator-space quantities; yaw and tendon transmissions have different coordinate semantics. Their aggregate and the numerical effort gate are implementation heuristics, not a common calibrated joint-torque or physical-force threshold.

The leg score is $S_{L}={\mathrm{clip}}_{\left[0,1\right]}\left(0.70F_{L}+0.15T_{L}+0.10M_{L}+0.03K_{L}+0.02I\right)$. The bounded tracking component $T_{L}$ normalizes mean command-to-transmission discrepancy by control-range spans, with floor 0.04 and full scale 0.32. $M_{L}$ measures lack of positive transmission velocity toward the activation target, with numerical full scale 0.5. $K_{L}$ compares finite-difference joint positions with measured joint velocities, normalized by the larger of the velocity norm and 1; it is zero at the first sample. The shared IMU component *I* is the clipped maximum of the relative acceleration-norm departure from 9.81 m/s^2^, gyro norm divided by 5 rad/s, and normalized upright deficit below 0.8. Toe force is logged but does not enter the score.

Entry requires $S_{L}\geq 0.58$ for five consecutive control samples; clearing requires $S_{L}\leq 0.24$ for 15 consecutive samples. These thresholds and weights are retained from development, not tuned to the main outcomes. Context components without an effort residual contribute at most 0.30 and cannot independently trigger entry. With synchronized, unsaturated measurements and sufficient expected effort, a 50% uniform strength reduction can yield $F_{L}\approx 0.5$, contributing only 0.35 to the score; partial faults can therefore remain undetected. That is a sensitivity limitation to report, not a failed trial to discard or a reason for outcome-dependent threshold adjustment. The complete component definitions are implemented in ResidualFaultDetector.update and ResidualDetectorConfig in hera_v2/physics.py; each trial records the detector configuration in its specification.

Measurements must refer to one post-integration state. A development audit identified a mismatch between refreshed activation/transmission quantities and stale force-dependent fields after the environment step. The implemented correction calls physics.forward() after env.step to recompute force and sensor fields without advancing time, position, velocity or activation. The completion timestamp is taken after synchronization, so its computational cost remains included in the measured latency. New records identify this path as mj_forward_post_integration. Pre-main regression tests verify unchanged integrated state, dynamic nominal-effort agreement for healthy channels, half-strength residual consistency and enabled force-limit handling; the correction is documented in DEVIATIONS.md. A fresh corrected-source pilot qualified the implementation before main execution. These implementation checks are not main-campaign outcomes, and pilot observations are excluded from the main analysis.

Six profiles cover complete failure (strength zero), partial failure (strength 0.5), healthy operation, complete failure with measurement noise, healthy operation with the same noise specification, and healthy operation with a lateral 40 N push lasting 0.2 s. Every profile crosses all four legs and phase offsets 0–19 with both methods. The actual reference phase is checked in the saved trace. Each profile–phase–leg cell has one seed and initial yaw, matched between methods; this is a finite leg–phase–seed grid, not independent stochastic replication or an intervention isolating phase alone.

Noise is added only to the copied detector measurements, not the outcome measurements: the independent zero-mean Gaussian effort noise with per-channel standard deviation is $0.05\max(|{\hat{f}}_{i}|,8)$, joint-position noise 0.002 rad, joint-velocity noise 0.02 rad/s, gyro noise 0.01 rad/s, and acceleration noise 0.05 m/s^2^. Activation and transmission position/velocity remain noiseless. This is consequently a partial sensor-noise sensitivity test, not comprehensive noisy proprioception. Paired methods share the deterministic noise seed; trajectory-dependent effort scale can still differ. These are synthetic stress settings, not measured hardware specifications. The push acts along the initial-body lateral direction and is not labeled an actuator fault. Responses to healthy and push conditions remain false actuator-fault responses under that labeling.

Physical eligibility uses the final 50 prefault samples: an upright measure of at least 0.95 in at least 95% of samples, maximum gyro magnitude of at most 0.50 rad/s, and minimum torso height of at least 0.35 m. Eligibility, detector correctness, reflex application and physical outcomes are separate fields. All trials, including ineligible ones, remain accounted for.

The operational fixed-horizon safety criterion requires eligibility, at least 50 postreference samples, no five-consecutive-sample fall (upright below 0.30 or height below 0.18 m), upright of at least 0.80 in at least 95% of postreference samples, and simultaneous upright of at least 0.80 and height at least 0.30 m in at least 95% of the final 50 samples. Healthy conditions use the same 3–10 s evaluation window. This is not a time-to-recovery endpoint. Continuous measures include minimum upright/height and $\sum_{k}\max\left(0,1-u_{k}\right)\Delta t$, as well as separately defined movement and displacement. Velocity magnitude is not forward mission progress.

A realtime audit uses phase offsets [0,2,5,7,10,12,15,17], all legs, and both methods. Absolute-deadline pacing and control lateness are recorded. With fault injection before control interval *k* and detection after interval *j*, simulation delay is (j+1−k)Δt. Omitting the +1 would understate this delay by one 20 ms control interval; this correction is separate from post-step force synchronization. Wall-clock timestamps are retained separately. Only paced audit/integration runs, not offline throughput, support the wall-latency descriptions.

### 3.5. Study C: Live Integration and Failure Containment

The methods are local_only, the three scheduler methods, and backend_failure. All use an identical immediate local response. Eight randomized complete batches cross four legs and all five methods. Each run lasts 12 s, with a physical fault at 3 s, incumbent budget 2048 tokens, and a supervisory deadline 9 s from the detector timestamp. A mission first-frame barrier starts the plant; failed backend start still permits the local plant run. Mission activity at detection is recorded rather than assumed.

The accepted canonical support reaffirmation is copied on a subsequent control tick and requests at most 50 control steps (1 s). If that duration is completed before the fixed 12 s observation horizon, control returns to the local fallback. A late application can instead be truncated by the trial horizon; requested duration, observed applied steps and observed expiry are therefore reported separately. The horizon is not extended to make a late command complete. Applied array, source, step, control-start timestamp, and payload identity are audited. The deliberately injected backend_failure condition raises an emergency-stream connection error after real mission inference; it is not a naturally observed server failure. local_only has no supervisor request, so its supervisor acceptance/application endpoint is not applicable rather than a failed request. Physical outcomes and local timing remain applicable to every method.

### 3.6. Follow-Up Studies and Retrospective Audits

The main campaign and its detector settings remain unchanged. After reviewing its results, we prepared separate source snapshots and manifests for a 560-trajectory degradation-warning evaluation and a 32-trial throughput comparison. The 592 additional trials are not an expansion of the original fixed matrix or a pooled inferential sample. Previously observed main results serve as development knowledge for the extension. Development trials are retained but excluded from both follow-up result sets; settings are not retuned after held-out outcomes are inspected.

#### 3.6.1. Separate Degradation Warning Without Control Intervention

The original severe-fault detector retains its 0.58/5-sample entry and 0.24/15-sample exit rules. A separate warning evaluates the sustained discrepancy between the same nominal predicted effort ${\hat{f}}_{i}$ and measured effort $f_{i}$, without access to the injected leg, strength or fault flag. In the most recent ten control samples, let $J_{L}$ contain samples whose leg-wise nominal effort sum is at least 8. When all ten temporal slots exist and $|J_{L}|\;\geq \;8$, the warning ratio is

(3) $$R_{L}=\frac{\sum_{j\in J_{L}}\sum_{i\in L}\left|f_{i}\left(j\right)-{\hat{f}}_{i}\left(j\right)\right|}{\sum_{j\in J_{L}}\sum_{i\in L}\left|{\hat{f}}_{i}\left(j\right)\right|}.$$

This is a ratio of accumulated discrepancies to accumulated expected effort, not an average of per-sample ratios. The actuator-space effort gate retains the same heuristic, mixed-transmission semantics as the original detector. Warning entry requires three successive ready windows with $R_{L}\geq 0.30$; clearing requires ten with $R_{L}\leq 0.15$. Insufficient excitation resets the counters but does not clear an existing warning: it is missing diagnostic evidence, not recovery. The warning is not a calibrated estimate of fault severity.

The window, excitation gate and thresholds were declared before the development pilot. A development-only comparison lowered the original score’s entry threshold to 0.38; its results are disclosed below but it was not selected as a held-out comparator. Two preserved 56-trial development runs used the same settings and seed 2026190801. The second qualified for strengthened audit tooling; it did not change the warning rule or selectively repeat failed scientific conditions. Qualification required all 56 records, unchanged control/source, detection of all 32 faulty trials by the severe-or-warning union within one simulated second, and no healthy, prefault or wrong-leg onset.

The subsequent fixed evaluation crosses seven profiles, four legs and 20 phase offsets (0–19), with master seed 2026290801 and a globally shuffled execution order. Profiles comprise the original six, plus half-strength faults with the same synthetic measurement noise. Seeds for the plant and noise are derived separately from the profile, phase and leg. Profiles therefore do not form matched noise/severity interventions. New seeds are a fresh conditional test on the same model and host, not unseen robots or wholly unseen phases. Each ten-second trajectory has 300 preliminary settle intervals and 500 measured control intervals, with a fault/reference at three seconds. The old severe detector, new warning and their union observe the same copied measurements in the shadow: every control action remains nominal trot. A warning neither activates the persistent local support action nor sends a language-service request. Correct post-injection onsets, misses, all other-leg/prefault/healthy onsets, clearing, and active duration are retained. Simulation-time detection delay uses the same (j+1−k)Δt convention as Study B.

#### 3.6.2. Matched Inference-Concurrency Throughput: Experimental Design

Eight temporal blocks each contain healthy-clean and complete-fault-clean local-reflex trajectories with inference idle and active, giving 32 trials. This comparison uses the original detector and local-reflex controller without the new warning observer; it does not measure the warning’s computational overhead. The main seed is 2026090901; four separate qualification trials use 2026190901. The leg rotates across blocks and the phase offsets are [0,2,5,7,10,12,15,17]. Within each profile/block, arms share the seed, initialization, phase and 500 control steps; the four conditions are randomly ordered within each block. Both arms use the same resident model and identical 16-token warmup before environment construction. The existing parallel-one context-8192 backend is verified before and after each trial; no server is reconfigured.

After settling, the active arm starts a fixed 2048-token, temperature-zero mission request. Its first nonempty nonterminal content frame releases a barrier before control step zero. The idle arm makes no benchmark generation request during the measurement window. The primary unpaced wall interval extends from the first recorded control-start timestamp to the explicitly recorded end of the last control interval, before result aggregation. It includes stepping, post-step forward synchronization, detector/controller execution and in-memory tracing, but excludes reset, settling, first-frame waiting and disk persistence. The numerator is the completed control steps, not MuJoCo integration calls; actual control/integration timesteps are checked as 0.02/0.005 s.

Active qualification requires content before the first and after the last measured step, at least three in-window content frames, no bracketing content gap above one second, and no stream termination before the loop end. This establishes sustained client-observed output, not continuous GPU-kernel occupancy. Traces with timestamps removed must match between paired arms. Host process/GPU boundary snapshots and server evidence are retained outside the window. Other experiment and document-rendering workloads under our control are paused, but unrelated host applications are not stopped. All failed or insufficient-overlap attempts remain accounted for; no outcome-based retry is permitted. We report eight paired relative changes per profile, including adverse or opposite-direction cases, as descriptive same-host observations.

#### 3.6.3. Reanalysis of Existing Observations

Leg-level scheduler aggregation uses the original 288 manifested observations, matched by workload, temporal block, leg and seed. Means across the eight observations for each leg are reported without treating the legs as independent robots. The four superseded manuscript latency summaries are traced to the exact 48 trial identifiers in their preserved source exports and to original JSON summaries. Available application logs localize observable delay segments but do not reconstruct absent server-internal telemetry. These audits add no experimental trials; their numerical findings are presented in Appendix A and Appendix B.

### 3.7. Analysis and Integrity

Exact frozen manifest IDs determine membership. Missing rows are restored for accounting; attempts and artifact identities determine which completed run is adopted. Paired comparisons use a matching profile/load, block, leg, and seed. Scheduler and integration uncertainty summaries use 5000 batch-cluster bootstrap draws with seed 20260907 and are descriptive/exploratory. Eight same-host temporal batches do not establish population-wide generality. Offline physics reports exact finite-grid means, ranges, and paired counts without population-inference claims. No confirmatory significance tests or retrospective power claims are made.

Timestamps use perf_counter_ns, backed on Windows by QueryPerformanceCounter; nanosecond storage does not imply nanosecond accuracy. UTC is used for log correlation only. Artifact acceptance requires hashes of source code, event logs and plant traces, command-application linkage checks, raw-trace recomputation of physical endpoints, full trial accounting, and reproduction of saved analysis. In the original main campaign, independent detector replay starts from saved scalar scores and verifies debounce, hysteresis and classification; those traces do not retain every raw sensor channel needed to reconstruct the complete score formula. Dynamic simulator regression tests cover nominal-force consistency separately. The partial-fault follow-up additionally retains the raw sensor channels and nominal actuator model, enabling complete severe-detector replay and separately implemented warning arithmetic/state replay. Its 280,000 sensor records and 280,000 corresponding physical records are paired views of the same 560 trajectories, not independent sample sets.

## 4. Results

### 4.1. Campaign Accounting and Runtime Verification

Campaign main_001 completed on 7 September 2026 at 13:36:32 UTC (22:36:32 KST), with all 1472 manifested trials adopted and verified, no missing or infrastructure-error trials, and all 11 per-manifest analyses complete. The total parent-process elapsed time was 5121.54 s (85.36 min), including 179.82 s for 32 batch lifecycle operations and 11.00 s for automatic analysis. These are campaign costs, not response latencies or pure simulation throughput. The corrected 160-trial qualification pilot remains excluded.

The scheduler audit checked 288 trials; the physical/integrated audit checked 1184 trials and 608,000 raw control samples; and the independent integration audit checked 160 trials. Each reported zero audit errors. All 11 archived analyses were reproduced exactly. Server evidence covered 32 batches, 64 exact historical log prefixes, and all 448 LLM-study records, with no unresolved configuration checks. The qualified source/model identities are those stated in the Methods. Every physical record identifies forward-synchronized measurement. Recorded pre-main measurement corrections and the main nominal-trigger timing deviation are retained in DEVIATIONS.md; no main trial was discarded or rerun because of its outcome. The 32 injected transport failures are planned scientific interventions, not missing observations.

### 4.2. Scheduler Outcomes

All 288 scheduler trials completed with a fully validated command accepted within the 30 s deadline: 32/32 for every method–workload cell. The canonical-response and raw-timestamp audit reported no errors. Because every trial was accepted, deadline-penalized and accepted-only means coincide in this study; the two definitions are nevertheless retained in this report.

Table 3 summarizes all nine method–workload cells.

The independent event audit found that the nominal 250 ms mission-ready-to-trigger delay was not achieved exactly: observed intervals ranged from 219.3589 to 254.9305 ms, with mean 237.8150 ms; 270/288 trials were below 250 ms. The mission-active trigger requirement held in every trial. All response/deadline endpoints use the actual trigger timestamp, not the nominal delay. This timing deviation is retained without rerunning the trials; its unique cause was not established.

At incumbent token caps of 256, 1024 and 2048, respectively, the mean fault-to-validated-acceptance times were 0.4864, 0.4859 and 0.5120 s for preemption; 1.4762, 5.2396 and 11.1010 s for FIFO; and 0.5430, 0.5442 and 0.5751 s for two-slot serving. These are within-workload comparisons; whole-workload groups were executed sequentially with different group-specific seeds.

Paired preemption-minus-FIFO mean differences were −0.9899 s (descriptive 95% batch-cluster interval −0.9940 to −0.9856 s), −4.7537 s (interval −4.7627 to −4.7445 s), and −10.5890 s (interval −10.7249 to −10.3985 s) at the three respective caps.

The paired differences between preemption and two-slot serving were −0.05658 s (interval −0.05884 to −0.05448 s), −0.05830 s (interval −0.06268 to −0.05486 s), and −0.06304 s (interval −0.06983 to −0.05626 s). Negative differences favor preemption for this endpoint. Each contrast contains 32 paired leg/seed cases across eight temporal batches; these intervals are exploratory descriptions of the tested campaign, not deployment-general or confirmatory inference.

Leg-level reaggregation of these same 288 trials is reported in Appendix A. Mean validated acceptance favored preemption over both comparators in all 12 workload–leg cells; all 96 individual preemption–two-slot paired differences were negative. This descriptive consistency check adds neither new trials nor independent robot replications.

The ordering depended on the endpoint. Two-slot serving dispatched the emergency request earlier (workload-specific means 0.945–0.954 ms) than preemption (2.503–2.634 ms), and its first valid streamed frame also arrived earlier (50.358–53.131 ms versus 58.579–61.392 ms). This is the client-observed first-valid-frame endpoint, distinct from the first nonempty content and from server-internal execution. Nevertheless, full validated acceptance favored preemption. FIFO’s mean client-observed queue intervals were 984.655, 4704.577 and 10471.789 ms. These observations distinguish dispatch, valid-frame and complete-command delivery; they do not establish when internal GPU work stopped or a slot was released.

Every completed emergency response reported 88 generated tokens. FIFO mission responses completed with the respective 256-, 1024- and 2048-token counts, with metadata available for all 32 requests in each cell. The final mission status was canceled in the preemption and two-slot conditions, and their completion token metadata were absent. Absence of those metadata is not zero mission work. In particular, final two-slot cancellation includes trial cleanup and does not imply that its emergency request required same-lane preemption or that mission completion was evaluated.

At the retrospective 1 s deadline, acceptance was 32/32 for both preemption and two-slot serving and 0/32 for FIFO at every workload. FIFO reached 32/32 by 2 s at the 256-token cap, by 10 s at the 1024-token cap (0/32 at 5 s), and by 30 s at the 2048-token cap (0/32 at 10 s). These fractions re-evaluate the same observed 30 s deadline trials; they are not new timeout experiments. Perfect acceptance at 30 s does not establish equivalence or zero failure risk.

**Figure 2:**
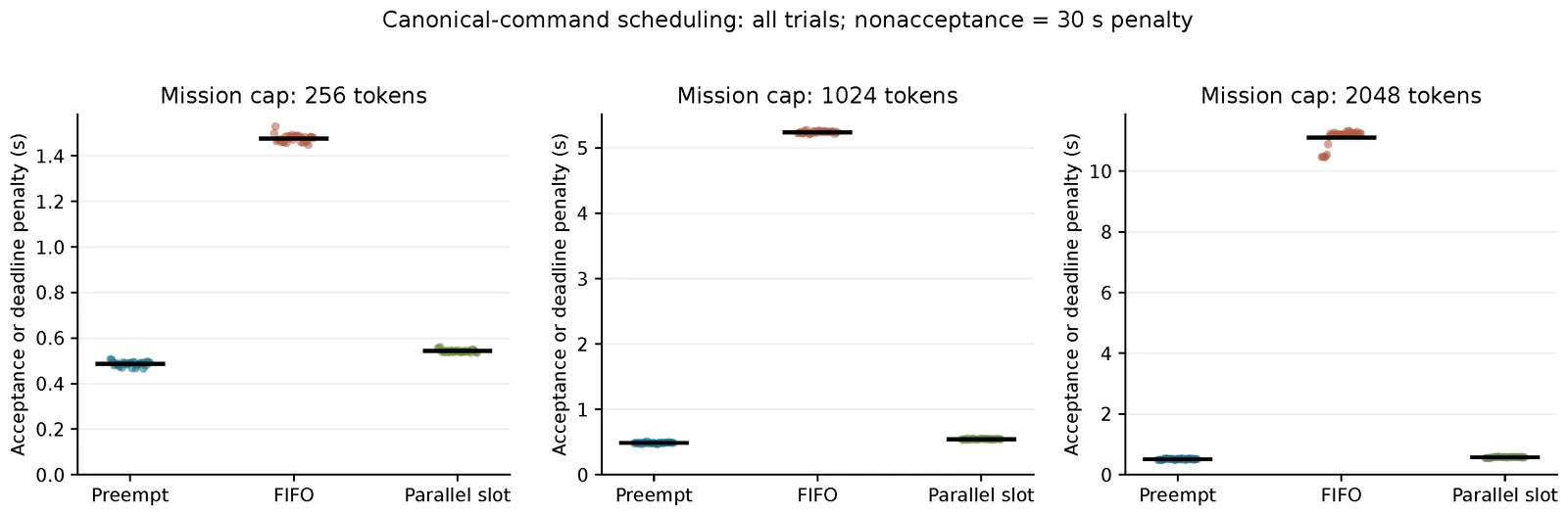
Client-observed validated-command latency by workload and scheduler. Comparisons are paired within workload; complete workload groups use different seeds and execute sequentially. The all-manifest deadline penalty is 30 s; none was needed because all 288 commands were accepted.

**Figure 3:**
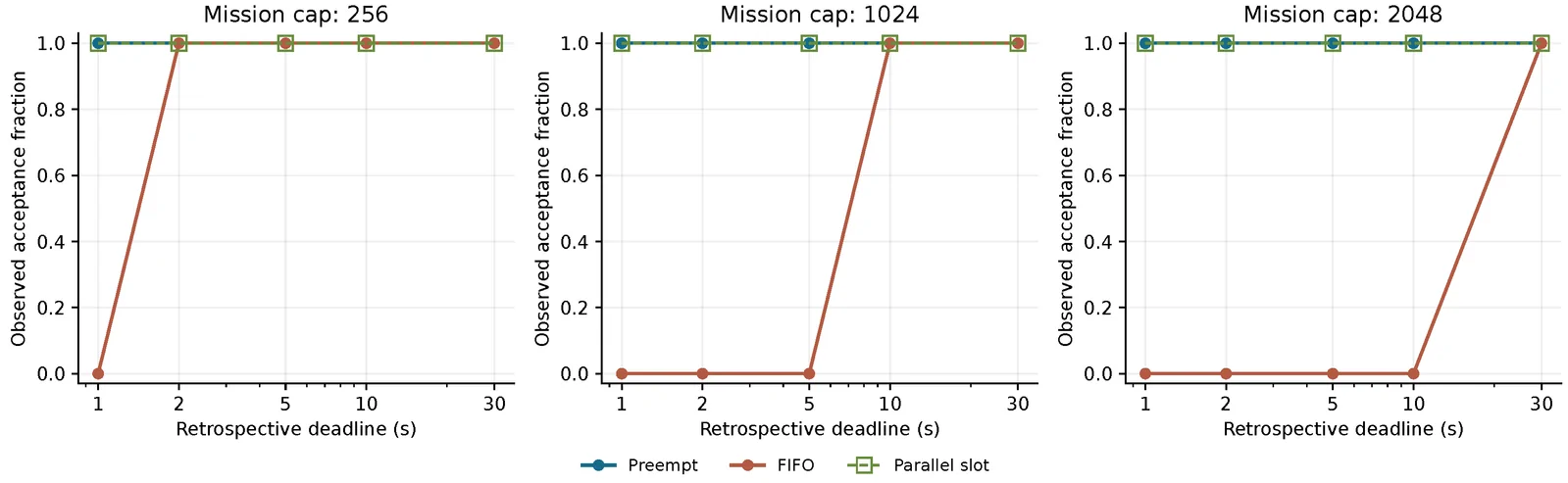
Retrospective deadline sensitivity from the same 30 s deadline observations; these are not new timeout experiments. Preemption and two-slot serving coincide at 100%; distinct markers expose the overlap. All manifested trials remain in each denominator.

Figure 2 and Figure 3 show the observed latency distributions and their retrospective deadline sensitivity.

### 4.3. Detector Coverage and Healthy Exposure

All 960 offline trials were eligible and physically evaluable, with 80 verified trials per profile/method and complete actual reference-phase coverage 0–19 for every leg. The selected-leg complete fault was correctly detected in all 160 clean and all 160 noisy offline trials. In contrast, every half-strength fault was missed (0/160 detected), despite satisfying the physical criterion. Its injected-leg score never reached 0.58 in the 56,000 postfault samples: the observed miss reflects no threshold crossing, not merely an insufficient consecutive-sample run. The separately paced audit and integrated conditions added 64 and 160 correctly detected complete faults, respectively: these were 544/544 complete-fault detections overall, not 704/704 detection of all fault severities. No prefault or other-leg false trigger was observed. These are outcomes of the fixed model-matched detector, not general diagnostic sensitivity.

The healthy-clean, healthy-noisy and healthy-push profiles each contributed 160 trials and 1600 simulated robot-seconds of observation: 480 s before and 1120 s after the 3 s reference. Total healthy exposure was therefore 4800 robot-seconds, excluding stand–settle time and without multiplying exposure by the four monitored legs. Every profile/method cell contributed 80 trials, 800 s of exposure and zero false events, false-trigger trials or false local-response onsets. False-response duty was zero; first-trigger times were censored at the 10 s horizon. True-positive detection and true-detection latency were N/A for these healthy conditions. The zero observed events under this limited synthetic exposure does not establish zero deployed false-alarm risk.

### 4.4. Finite-Grid Physical Outcomes and Realtime Timing

Every offline trial met the operational fixed-horizon safety criterion (960/960), with no falls; each profile/method was 80/80. This ceiling yields zero paired safety differences and does not show that local support is necessary to remain upright. Table 4 reports the separate detector and continuous endpoints. For complete clean faults, the mean local-minus-no-reflex upright deficit was −0.12194 s: 72 of 80 matched cells improved and eight worsened. For complete noisy faults, the mean difference was −0.11946 s: 70 improved and 10 worsened. There were no ties in either full-fault grid; all 80 pairs tied in each partial or healthy profile because no local response was triggered. These are exact finite-grid counts, not population significance tests.

In the complete clean condition, the minimum observed upright was 0.89983 with local support and 0.86984 without it; the minimum height was 0.40945 and 0.37695 m, respectively. In the noisy condition, minimum upright was 0.89142 and 0.87353, and minimum height was 0.40801 and 0.38138 m. These are minima across trials, not means. Mean terminal speed fell from 0.49004 to 0.10222 m/s in the clean grid and from 0.49198 to 0.10240 m/s in the noisy grid. A lower upright deficit therefore coexisted with reduced motion; velocity magnitude is not forward recovery or task completion. Cross-profile differences are not matched noise/severity effects.

**Figure 4:**
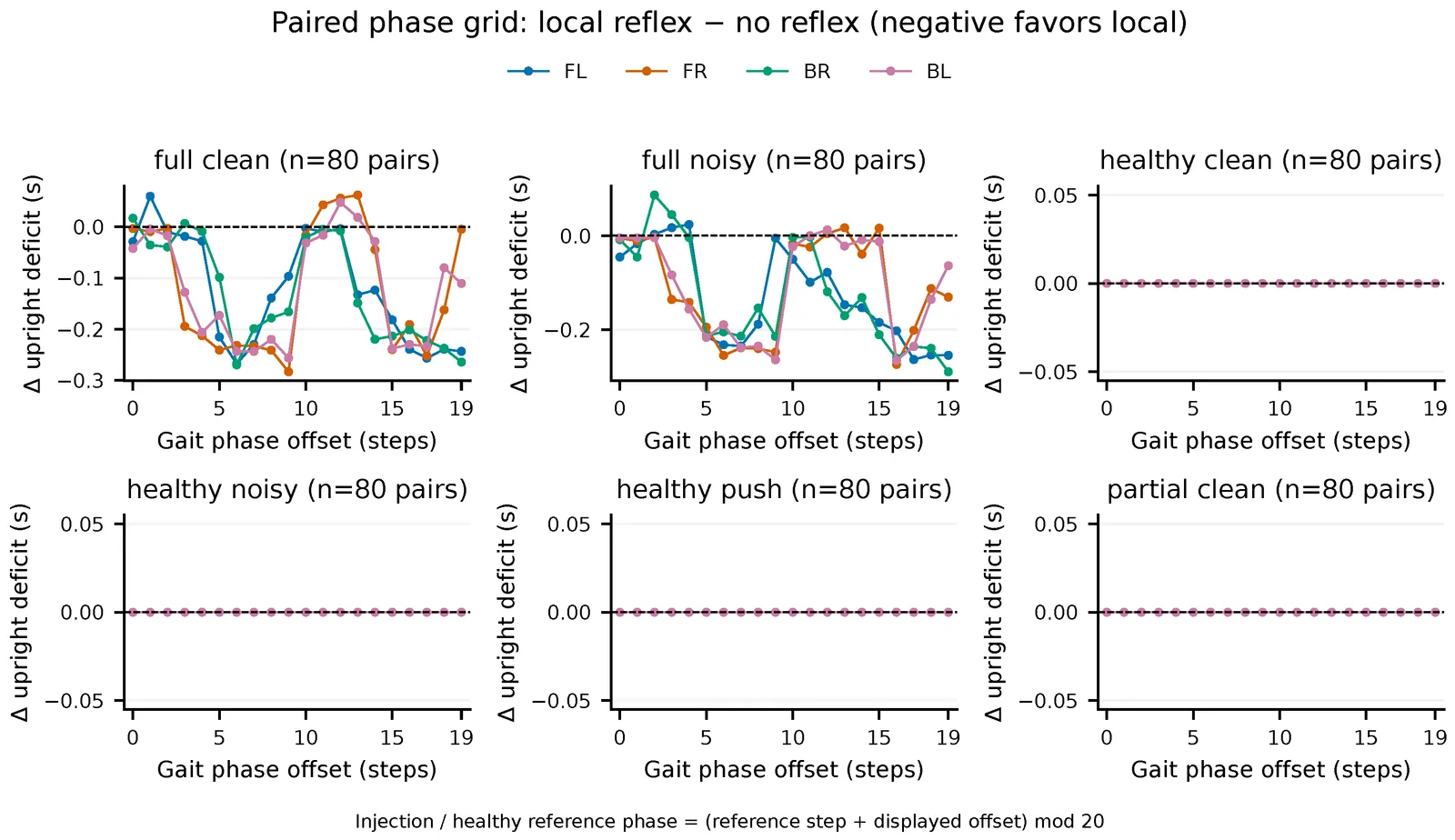
Paired local-minus-no-reflex upright-deficit differences across the finite leg–phase–seed grid. The black horizontal dotted line marks zero paired difference. Negative values favor local support for this continuous endpoint; adverse cells remain visible. The plotted configured phase offset differs from the actual reference phase by the recorded reference-step offset modulo 20. Each cell has one seed/yaw, so variation is not attributable to phase alone. No population-inference interval is attached.

Figure 4 displays the paired continuous differences, including adverse cells.

All 64 realtime-audit trials were eligible, correctly detected and physically safe (32 per method). Detection required 100 ms of simulated time in every case. Mean measured wall detection delay was 79.409 ms with local support and 79.570 ms without it; the median values were 79.463 and 79.629 ms. Local detector-to-action delay had mean 19.514 ms, median 19.491 ms and range 18.707–20.161 ms (n=32). Its reported simulation delay was zero because detection occurs at the end of one control interval and application begins at the next boundary; the wall delay was not zero. Adding the two observed wall intervals gave mean fault-to-local action 98.922 ms, versus 100 ms in simulation. Different pacing and integration conventions explain why the simulated and wall intervals were not interchangeable.

Median per-trial maximum pacing lateness was 1.169 ms (range 0.858–3.958) with local support and 1.190 ms (0.861–5.642) without it. The observed worst value was 5.642 ms, which was not a hard-realtime bound. No-reflex action timing was N/A. Table 5 retains timing denominators and clock distinctions; offline processing speed is not used as response-latency evidence.

### 4.5. Live Command Application and Local Independence

All 160 integrated trials completed and passed independent identity/timing audits: 32 for each method, all eligible, correctly detected and physically safe, with no fall. The mission was active at detection in all 128 inference-bearing conditions; mission activity was N/A for the 32 local-only controls. Local detector-to-action means ranged from 19.366 to 19.744 ms across the methods (Table 6), while delayed or deliberately failed supervision did not prevent local application. The worst observed integration pacing lateness was 9.650 ms across all 160 trials; this remains a host-specific observation.

Every method had the same mean upright deficit (0.147781 s), median deficit (0.001460 s) and mean terminal speed (0.089186 m/s), with identical paired physical outcomes. The large mean–median difference reflects heterogeneity among the finite scenarios, not added supervisory benefit. Local-only and injected-failure records are retained in these physical denominators.

All 96 normal supervisory requests were accepted, applied for all 50 requested steps, and followed by an observed return to local fallback: 32/32 for preemption, FIFO and two-slot serving. No main command was horizon-truncated, and no expiry was horizon-censored. The mean detector-to-acceptance times were 493.627, 7144.086 and 556.898 ms, respectively; the mean acceptance-to-application times were 29.810, 30.027 and 28.865 ms. The corresponding detector-to-actual-supervisor-application means were 523.437, 7174.113 and 585.763 ms. These are conditional timing summaries with n=32 per normal method; the detector start differs from Study A’s external trigger. The 32 injected emergency transport failures produced no accepted or applied supervisor command as intended, while local-only had no supervisor request. Duration/expiry and supervisor timing were N/A in those two conditions, not failed 50-step holds.

The 160-trial command audit found no stale, pre-validation, mismatched or unaccepted application. For example, the first-batch BL preemption trial with seed 1573589318 accepted its command 523.108 ms after detection, and first applied it 36.309 ms later, on step 182. All 50 steps through step 231 were observed, followed by local fallback; 26 local-action steps had already occurred before acceptance. This event-to-command-to-control linkage is the integration result. Since the command reaffirms the existing array, it does not demonstrate additional LLM physical benefit.

**Figure 5:**
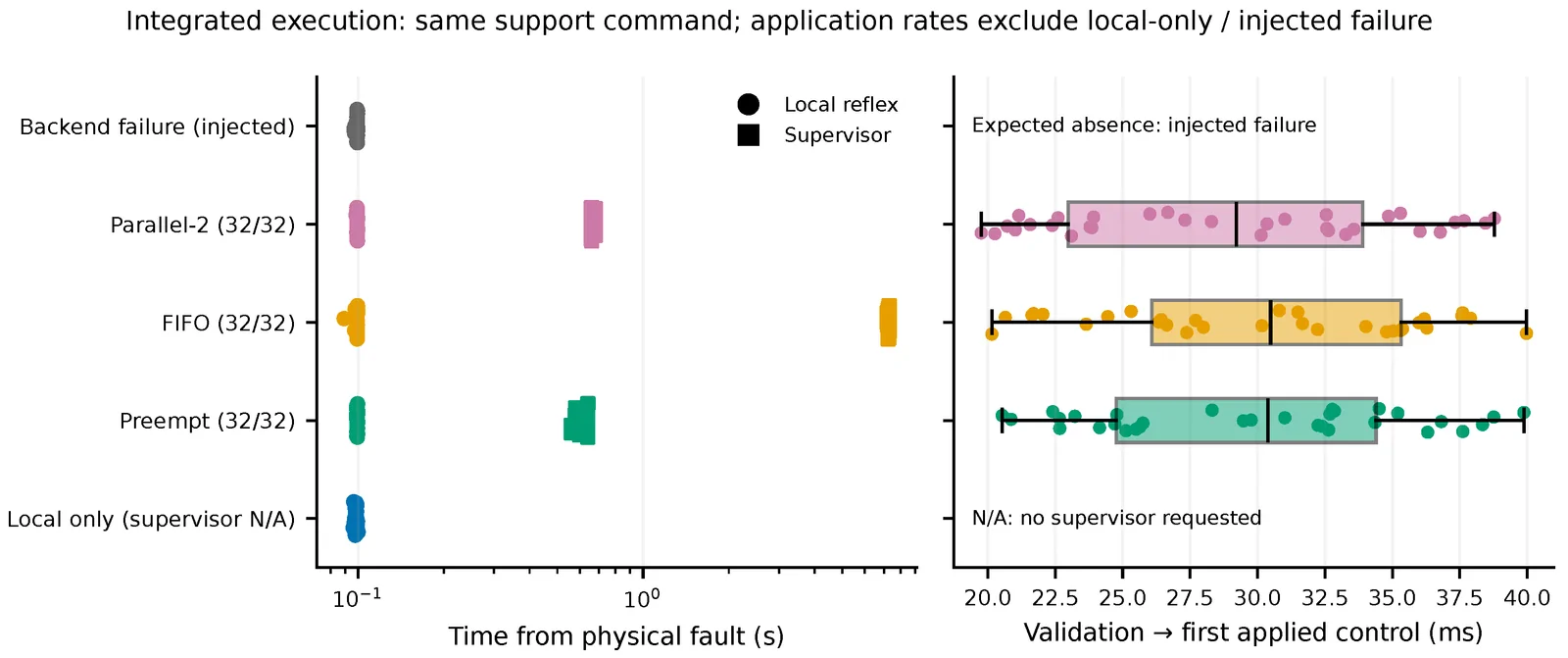
Observed integrated application timing and command lifecycle from the final main traces. Local-only supervision is N/A, and the injected transport-failure condition expects no supervisor command. Every normal supervisory request completed its 50-step hold and had observed fallback; no main hold was horizon-truncated. The supervisor applies the same support array as the independent local response.

Figure 5 summarizes the observed integrated application timing and lifecycle.

### 4.6. Separate Held-Out Degradation-Warning Evaluation

All 560 manifested trajectories completed without omission or selective rerun. Independent checks matched each specification to its manifest, verified source and artifact identities, reproduced the report aggregates, and replayed all 280,000 sensor records against 280,000 corresponding physical records. The original severe-detector implementation and independently implemented warning arithmetic/state replay agreed with the recorded outputs. Control remained a nominal trot throughout all trajectories. These checks establish recorded-data consistency, not correctness under untested plant models.

Table 7 separates the original severe-detector outcomes from the warning outcomes on the same new trajectories. The severe detector missed all 80 clean and all 80 noisy half-strength faults; the warning detected all 160. The mean warning delays were 161.0 and 155.5 ms, respectively, with a maximum of 200 ms in each profile, all in simulated time. Both paths detected all 160 complete faults. For complete clean/noisy faults, the mean warning delays were 105.0/101.75 ms versus 100 ms for the severe detector; the union’s means were 93.0/91.75 ms. Thus, the warning alone was not uniformly faster than the severe pathway. Healthy-clean, healthy-noisy and healthy-push each contributed 80 trajectories and 800 robot-seconds, with no onset in either path. No prefault or wrong-leg onset occurred in any of the 560 trajectories. Exposure excluded settling and was not multiplied by the monitored legs.

The held-out comparison demonstrates sensitivity to the specified half-strength faults while preserving the severe pathway and its control semantics. It does not establish a physical benefit from acting on a warning. The development-only score threshold of 0.38 also detected all 16 development partial faults without observed false onsets at a mean simulated delay of 100 ms; the new warning’s corresponding development means were 150 and 142.5 ms. Consequently, superiority over a simple threshold reduction was not supported. That contrast was not measured in the 560-trial evaluation, and absent values must not be interpreted as zero-event results. The reason for a separate warning is to avoid equating degradation notification with a persistent support intervention, not a demonstrated advantage over all alternative detectors. The original main-campaign result of 0/160 detections remains unchanged.

### 4.7. Matched Inference-Concurrency Throughput: Results

All 32 main trials qualified, and all 16 paired scientific traces matched exactly after removing the timestamps. Each trial completed 500 control steps with the checked 4:1 integration/control relationship. Active windows contained 51–62 nonempty content frames; the largest bracketing gap was 50.7746 ms. Model identity, context, parallelism and backend process identity were consistent across all 64 before/after observations. The raw-window, stream-overlap, trace and 64 server-log-slice audits passed. A preserved first qualification attempt failed all four conditions before physical measurement because the validator expected legacy backend command-line options; correcting identification was followed by a fresh successful four-trial pilot. Neither pilot was pooled with the main comparison.

Table 8 summarizes the paired throughput comparison. The mean paired throughput change with concurrent inference was −9.53% for healthy-clean and −6.53% for complete-fault-clean local reflex. Medians were −5.16% and −7.40%; the respective ranges were −31.61% to −4.23% and −14.18% to +4.79%. All eight healthy pairs were slower with active inference, but two of the eight complete-fault pairs were faster. These are means of block-wise ratios, not ratios of arm means. No large or opposite-direction observation was removed.

The measurement windows were only 0.242–0.359 s. Boundary snapshots showed CPU activity by unrelated applications, and those boundaries also included setup and request cleanup. They did not identify which external work overlapped a particular short control window or caused an individual slowdown. The observed comparison therefore includes service, streaming-client and shared-host effects. It is neither an isolated GPU-cost estimate nor a retrospective causal explanation of the superseded 4800 versus 5290–5530 steps/s values. It also does not measure unnecessary-interrupt cost, energy consumption or sustained mission throughput.

## 5. Discussion

### 5.1. What the Experiments Can Establish

Study A measured client-observed delivery of a fully validated, predetermined command. All 288 trials met the 30 s deadline. Within each workload, preemption reduced full-command acceptance time relative to FIFO and was approximately 0.057–0.063 s earlier than two-slot serving; both preemption and two-slot serving met the retrospective 1 s deadline in every trial. Two-slot serving was nevertheless dispatched earlier and produced an earlier valid streamed frame, an endpoint distinct from nonempty content. Thus, the result is endpoint-specific, not evidence that cancellation improves every stage or is necessary whenever parallel capacity is available. All emergency outputs reported the same 88 generated tokens, but interrupted mission work was not fully quantified because completion metadata were absent for cancelled requests. Neither short command-delivery time nor structured serialization establishes fault diagnosis or physical recovery.

Study B separates detector behavior from physical outcomes. In the original main campaign, complete faults were correctly detected across the grid, whereas all 160 half-strength faults were missed. No false trigger was observed during 4800 simulated robot-seconds of healthy exposure. Every original physical trial met the fixed-horizon safety criterion, including undetected partial faults and no-reflex controls. Local support lowered the mean upright deficit in complete-fault conditions, but worsened the eight clean and ten noisy paired cells and reduced terminal speed. These outcomes do not establish a safety-success advantage or recovery of locomotion. Detection, correct application and physical success answer different questions. Neither a zero observed failure count nor a zero-width resampling interval proves equivalence or zero risk.

The separate warning follow-up addresses the detector’s specific sensitivity gap: on the same new half-strength observations, detection changed from 0/160 for the unchanged severe detector to 160/160 for the warning. The original miss count remains part of the evidence. The warning has no control authority, so its successful detection cannot be used to claim improved stability, locomotion or task recovery. A simple lower-threshold development contrast also succeeded and was faster in that pilot. The supported contribution is therefore conditional detection coverage with a separate degradation notification, not a superior general diagnostic algorithm. Any proposal to act on partial-fault warnings needs a separate physical comparison that includes unnecessary intervention and movement loss.

Study C audited measured events and independently executed local responses across 160 trials, with validated supervisory application in all 96 normal-request trials. Local action followed detection in approximately 19–20 ms on average across methods, including injected transport failure. All 96 normal supervisory requests were applied for 50 steps and expired to the local fallback; none was horizon-truncated in the main campaign. The identical physical outcomes across all five conditions are consistent with the shared action, and are not evidence that language inference adds physical value. Successful application establishes bounded closed-loop integration and containment of the tested inference failure, not LLM-discovered stabilization, unrestricted replanning or additional physical benefit beyond local-only control. Those stronger claims require meaningful supervisory choices and an appropriate non-LLM comparator.

### 5.2. Scheduling, Resources, and Timing Boundaries

Preemption trades interruption of mission work for delivery of another request. The two-slot comparison uses the same GPU; it is neither an independent emergency processor nor a hard priority reservation. Cancellation completion and streamed output are client observations. Server logs verify the loaded model and runner configuration, but without internal request-identity timing, they do not establish the instant at which GPU work stops or a slot becomes free. Backend- and workload-specific results must not be generalized to arbitrary serving systems.

The service-level cost of deliberately unnecessary interrupts, including interrupted mission work, regeneration, and unnecessary stopping, was not separately measured. No false triggers were observed in the specified healthy tests, but this does not establish the cost of an unnecessary scheduler interruption.

The separate matched throughput comparison now provides direct current-host observations of concurrent inference service activity: mean paired throughput was lower in both tested profiles, while two complete-fault pairs showed the opposite direction. Short windows and observed external application activity limit precision and attribution. HTTP streaming, client buffering and shared-host effects are included, so the result is not an isolated GPU cost. It does not retrospectively explain historical processing rates. The audit in Appendix B instead traces superseded response-time values to their actual batches and separates an application-observation delay from the logged inference interval; the missing server-internal cause remains unresolved.

The local response has a deterministic software decision path, but Python threads, an operating-system scheduler, and a shared GPU/CPU host do not provide a verified hard-realtime guarantee. We report control lateness and both simulation and wall-clock delays. Offline grid throughput is not a substitute for wall-clock latency, and unpaced simulated time is not a physical realtime experiment. The corrected injection-to-observation convention includes the integration step before the detector can observe its effect.

Even the scheduler’s configured 250 ms pretrigger wait differed from its observed event interval. Inexact event-loop or host timer scheduling is a possible contributor, not an isolated causal finding. Reporting achieved event times and defining latency from the actual trigger avoids treating a software delay setting as an exact wall-clock intervention.

### 5.3. Biomimetic and Physical Limitations

HERA is bioinspired at the level of temporal separation and local response, not a validated neural architecture. Biological references justify the design questions, while the software cancellation pathway remains an implementation analogy. The support array, nominal-effort residual, and synthetic noise have no demonstrated correspondence to identified neural circuitry or calibrated hardware sensors. Persistent actuator-strength reductions and a lateral push broaden the simulation tests but do not exhaust contact uncertainty, communication faults, sensor bias, friction variation, fatigue, or compound failures. Operational upright/height thresholds over a fixed horizon do not certify stability, locomotion recovery, hardware safety, or mission completion.

The original residual detector is a model-matched simulator nominal-force consistency test, weighted primarily toward actuator-model mismatch, with fixed entry threshold 0.58 and five-sample debounce. It missed all 50% strength losses in the main campaign and in the new shadow comparison. The added warning improves coverage of those specified losses, but both mechanisms rely on the same nominal plant model. Parameter uncertainty, different degrees of degradation and hardware calibration remain untested. Activation- and transmission-state inputs remain noiseless in the specified synthetic noise test. Post-step field synchronization and the one-control-interval latency correction are measurement-integrity requirements, not main-outcome threshold tuning. Earlier pilot records are preserved and excluded. Original main-trace replay verifies the detector state machine from saved scores; the new shadow records additionally support full-sensor severe replay and independently implement warning replay. Neither audit removes the shared model assumptions.

### 5.4. Inference, Reproducibility, and Future Work

The main matrix was fixed before execution, with implementation pilots excluded. Temporal batch-cluster intervals summarize variability in the tested scheduler/integration campaign; eight batches on one host are not independent deployments. The offline grid is an exhaustive finite design, not a random sample of robots or environments. It supports descriptive coverage and paired differences, not population-generalizing significance tests. All 1472 manifested trials were retained, applicable artifact audits reported zero errors, and all 11 saved analyses were reproduced exactly. The pre-main measurement corrections and the observed nominal-trigger timing deviation are disclosed rather than hidden through replacement or selection. These computational checks establish the consistency of the recorded evidence, not freedom from every modeling or implementation error.

In the original main campaign, randomization is restricted to method/leg order within batches. Whole-workload and physical-profile groups execute in a fixed sequence and use label-specific scenario seeds. Consequently, method contrasts are paired within a workload or profile, whereas clean–noisy, severity and cross-workload comparisons are not matched interventions. Each physical leg–phase cell also has one seed/yaw, so apparent phase variation cannot be attributed to phase alone. The follow-up shadow matrix randomizes order across its 560 cells, while the throughput comparison randomizes four conditions within each of eight blocks. These separate designs improve specific comparisons but do not convert either the original or new observations into independent robots or deployments. In particular, the small difference between clean and noisy warning-delay means is not an isolated beneficial effect of noise.

Future work should separately evaluate informative supervisory decisions, task-level recovery, decisions to act on degradation warnings, calibrated noisy sensing, hardware execution, and additional backends. These are unresolved questions, not capabilities implied by schema-constrained output. Structured decoding and application-side validation constrain an interface [16]; they do not establish general semantic reliability or safety. The updated HERA archive at https://doi.org/10.5281/zenodo.22667761 preserves the original 1472-trial deposit unchanged and includes the separate 560-trial warning and 32-trial throughput studies, their source and raw records, and portable replay and analysis tools. New leg-level and historical audit materials are included; historical inputs remain bounded extracts, not complete legacy raw traces. Verification scopes and the distinction from the public code repository are specified in the Data Availability Statement. Access and computational reproducibility do not resolve the modeling or generalizability limitations above.

## 6. Conclusions

The fixed 1472-trial campaign supports separating local physical response from language-service scheduling. On the tested backend, preemption delivered a fully validated canonical command earlier than FIFO and modestly earlier than two-slot serving, although two-slot dispatch and first-frame arrival were earlier. The original model-matched detector identified complete faults, but missed every half-strength fault. In a separate 560-trial evaluation, the added effort-discrepancy warning detected all 160 new half-strength faults that the unchanged detector missed without observed false onsets in 2400 robot-seconds of healthy exposure. Since this warning had no control authority, the result establishes a bounded sensitivity improvement, not better recovery or superiority over simple threshold adjustment. A separate 32-trial comparison observed lower mean throughput with concurrent inference service activity on the current shared host. The original local support reduced the mean upright deficit while worsening some cells and reducing terminal speed; the safety ceiling and identical integrated actions preclude additional safety-success or LLM physical-benefit claims. These are finite simulation and host-specific results, not physiological validation, general diagnosis, autonomous recovery, immediate GPU release or hard-realtime hardware safety results.

## Figures and Tables

**Table 1:** Biological inspiration is separated from the implemented abstraction and its test. These mappings are design rationales, not physiological validation.

Inspiration	Engineering Abstraction	Evidence Required Here
Local sensorimotor response	Measurement-driven deterministic support command	Detector residuals, false responses, command and plant traces
Prediction/feedback discrepancy	Nominal-effort and proprioceptive residuals	Measured fault and healthy-condition performance; no cerebellar equivalence
Separation of temporal roles	Local control independent of a slower language service	Control timing during delayed or failed supervision
Context-dependent escalation	Event notification and bounded request preemption	Client-observed scheduling and accepted/applied command identity; no anatomical pathway claim

**Table 2:** Fixed main matrix. All 1472 manifested trials completed and passed the applicable artifact audits; completion is distinct from scientific success. The separately frozen follow-up studies are not part of this matrix.

Study	Fixed Crossing	Trials
A: scheduler	3 loads × 8 batches × 4 legs × 3 methods	288
B: offline physics	6 profiles × 20 phases × 4 legs × 2 methods	960
B: realtime audit	8 phases × 4 legs × 2 methods	64
C: integration	8 batches × 4 legs × 5 methods	160
Total	Separate implementation pilot: 160 trials, excluded from main	1472

**Table 3:** Canonical-command scheduling by incumbent token cap. Pen. mean includes each nonaccepted manifested trial at its 30 s deadline; accepted-only summaries are conditional. The 1 s fraction retrospectively reuses these trials, not a separate timeout experiment.

Cap	Method	*N*	Accepted	Pen. Mean (s)	Accepted Mean ±SD (s)	Accepted Median (s)	≤1 s
256	Preempt	32	32	0.486	0.486 ± 0.010	0.488	100.0%
256	FIFO	32	32	1.476	1.476 ± 0.016	1.478	0.0%
256	Two-slot	32	32	0.543	0.543 ± 0.006	0.544	100.0%
1024	Preempt	32	32	0.486	0.486 ± 0.009	0.486	100.0%
1024	FIFO	32	32	5.240	5.240 ± 0.015	5.243	0.0%
1024	Two-slot	32	32	0.544	0.544 ± 0.005	0.544	100.0%
2048	Preempt	32	32	0.512	0.512 ± 0.017	0.516	100.0%
2048	FIFO	32	32	11.101	11.101 ± 0.280	11.210	0.0%
2048	Two-slot	32	32	0.575	0.575 ± 0.013	0.579	100.0%

**Table 4:** Offline finite-grid outcomes. Detected denotes a recorded selected-leg postfault detection; healthy detection is not applicable (N/A). False-event parentheses count trials with at least one false trigger. Safe uses all manifested trials; deficit is the upright-deficit integral. These are finite-grid descriptions, not population-inference estimates.

Profile	Method	*N*	Safe/*N*	Detected/*N*	False Events(Trials)	Mean Deficit(s)
Full, clean	Local reflex	80	80/80	80/80	0 (0)	0.070
Full, clean	No reflex	80	80/80	80/80	0 (0)	0.192
Partial, clean	Local reflex	80	80/80	0/80	0 (0)	0.005
Partial, clean	No reflex	80	80/80	0/80	0 (0)	0.005
Healthy, clean	Local reflex	80	80/80	N/A	0 (0)	$1.75\times 10^{-6}$
Healthy, clean	No reflex	80	80/80	N/A	0 (0)	$1.75\times 10^{-6}$
Full, noisy	Local reflex	80	80/80	80/80	0 (0)	0.073
Full, noisy	No reflex	80	80/80	80/80	0 (0)	0.193
Healthy, noisy	Local reflex	80	80/80	N/A	0 (0)	$2.21\times 10^{-6}$
Healthy, noisy	No reflex	80	80/80	N/A	0 (0)	$2.21\times 10^{-6}$
Healthy, push	Local reflex	80	80/80	N/A	0 (0)	$5.49\times 10^{-4}$
Healthy, push	No reflex	80	80/80	N/A	0 (0)	$5.49\times 10^{-4}$

**Table 5:** Separate realtime physics audit. Detection starts at fault injection; local dispatch starts at detection. Parentheses count non-null observations, with no-reflex application N/A. Simulation and wall clocks are distinct: zero simulation delay at an adjacent tick boundary does not equate to zero wall latency. Maximum lateness is the largest observed trial maximum, not a hard-realtime bound.

Method	*N*	Detect Sim. Mean ms (*n*)	Detect Wall Mean ms (*n*)	Local Sim. Mean ms (*n*)	Local Wall Mean ms (*n*)	Max Lateness ms (*n*)
Local reflex	32	100.000 (32)	79.409 (32)	0.000 (32)	19.514 (32)	3.958 (32)
No reflex	32	100.000 (32)	79.570 (32)	N/A	N/A	5.642 (32)

**Table 6:** Integrated command delivery and observation. Full means all 50 requested ticks were observed; truncation retains the fixed horizon; expiry requires an observed subsequent local-fallback tick. Both delays start at the detector event and end at the relevant actual control tick. Parentheses give applicable timing counts. Local-only supervision is N/A; * zero delivery is expected under the injected emergency transport failure, with duration/expiry endpoints N/A. Canonical reaffirmation does not establish extra physical benefit.

Method	*N*	Accept	Apply	Full	Trunc.	Expiry	Local Mean ms (*n*)	Supervisor Mean ms (*n*)
Local only	32	N/A	N/A	N/A	N/A	N/A	19.366 (32)	N/A
Preempt	32	32	32	32	0	32	19.711 (32)	523.437 (32)
FIFO	32	32	32	32	0	32	19.744 (32)	7174.113 (32)
Two-slot	32	32	32	32	0	32	19.564 (32)	585.763 (32)
Transport failure	32	0 *	0 *	N/A	N/A	N/A	19.486 (32)	N/A

**Table 7:** Separate 560-trajectory shadow follow-up, with 80 observations per profile. Original denotes the unchanged severe detector; warning denotes the new sustained-effort path, not a control intervention. Detection is correct postfault localization; healthy detection and latency are N/A. Warning delays are simulated milliseconds, conditional on detection. Warning false counts include prefault, wrong-leg and healthy onsets; parentheses count affected trials. Healthy exposure totals 2400 robot-seconds, without multiplying by four monitored legs. The development-only 0.38 threshold contrast was not evaluated in this holdout.

Profile	Original Correct/*N*	Warning Correct/*N*	Warning Mean (Sim. ms)	Warning Max (Sim. ms)	Warning False Onsets (Trials)	Healthy Robot-s
Full, clean	80/80	80/80	105.00	160.00	0 (0)	0
Partial, clean	0/80	80/80	161.00	200.00	0 (0)	0
Healthy, clean	N/A	N/A	N/A	N/A	0 (0)	800
Full, noisy	80/80	80/80	101.75	160.00	0 (0)	0
Partial, noisy	0/80	80/80	155.50	200.00	0 (0)	0
Healthy, noisy	N/A	N/A	N/A	N/A	0 (0)	800
Healthy, push	N/A	N/A	N/A	N/A	0 (0)	800

**Table 8:** Separate 32-trial matched inference-service throughput follow-up (eight valid pairs per profile). Rates count completed control steps/s, not internal integration steps. Paired change is $100(r_{\mathrm{active}}/r_{\mathrm{idle}}-1)$ within each block; the mean of these changes is not the ratio of the two arm means. Physical traces matched within every pair. All eight healthy pairs and six of the eight full-fault pairs were slower with active inference; the two opposite-direction full-fault pairs remain included. This is a same-host service-concurrency observation, not isolated GPU cost or a retrospective explanation of historical throughput.

Profile	*n* Pairs	Idle Mean Steps/s	Active Mean Steps/s	Paired Mean Change (%)	Paired Median Change (%)	Paired Range (%)
Healthy, clean	8	2006.10	1812.87	−9.53	−5.16	[−31.61, −4.23]
Full fault, local reflex	8	1950.39	1819.08	−6.53	−7.40	[−14.18, +4.79]

## Data Availability

The HERA data, execution source and verification materials are publicly archived at https://doi.org/10.5281/zenodo.22667761 (accessed on 7 September 2026). This is an external research-data deposit, not journal-hosted supplementary material. The updated release embeds the original 1472-trial archive byte-for-byte, including 1184 physical and integrated control traces, and adds the separately frozen 560-trial warning and 32-trial throughput studies with their source, manifests, raw records and portable saved-data tools. These studies are not pooled. Development and pilot records are retained separately; historical audit inputs are bounded summary, CSV and log extracts, not complete legacy raw traces. Fresh-extraction verification of the updated release checked the public manifest, passed 48 release-adapter integrity/privacy tests, verified all 280,000 sensor and 280,000 physical rows of the warning holdout and all 32 throughput trials, and reproduced three follow-up tables and their numerical text. The original archive’s verification separately passed 76 regression tests and reproduced 11 main analyses and four main-result tables. These are saved-evidence checks, not additional experiments. The new public record and checksum file were accessed without authentication; the reported ZIP size and MD5 and published SHA-256 match the locally verified release. The public repository at https://github.com/goddongyoun/HERA_dataset (accessed on 7 September 2026) contains software and selected exports rather than all raw follow-up data. The project software is licensed under MIT, and the scientific data and figures under CC BY 4.0. Selected identifying metadata are masked, with original/public file identities recorded separately; scientific raw values and outcomes are unchanged.

## Appendix A. Leg-Level Scheduler Aggregation

The same 288 Study A observations were grouped by workload, method and leg, retaining all eight temporal blocks per cell. The 36 leg-level method means reproduced the preserved analysis. Every workload–leg cell favored preemption over FIFO and over two-slot serving for final validated acceptance. Table A1 gives the smaller, practically closer preemption–two-slot contrast. Averaging the four leg means reproduces the original workload-level comparison because the design is balanced. Leg aggregation addresses the repeated-measure presentation; it does not eliminate dependence induced by the shared host or create independent robot replications. Dispatch and first-frame endpoints retain their different ordering.

**Table A1:** Leg-level descriptive reanalysis of the existing 288 scheduler trials; no new scheduler observations. Each row contains eight paired temporal blocks. The difference is preemption minus two-slot fully validated acceptance latency within workload, block, leg and seed; negative values favor preemption for this endpoint. All 36 method–workload–leg means are shown; all 96 preemption–two-slot paired differences are negative. All trials shown are accepted. Four legs are not independent robots, and this table does not add a confirmatory significance test or reverse the earlier dispatch/first-frame advantage of two-slot serving.

Cap	Leg	*n* Pairs	Preemption Mean (s)	FIFO Mean (s)	Two-Slot Mean (s)	Paired Mean Difference (ms)
256	FL	8	0.4900	1.4703	0.5422	−52.197
256	FR	8	0.4837	1.4784	0.5444	−60.707
256	BL	8	0.4848	1.4686	0.5440	−59.275
256	BR	8	0.4871	1.4877	0.5413	−54.161
1024	FL	8	0.4828	5.2405	0.5454	−62.665
1024	FR	8	0.4857	5.2424	0.5462	−60.510
1024	BL	8	0.4854	5.2380	0.5410	−55.544
1024	BR	8	0.4896	5.2375	0.5441	−54.498
2048	FL	8	0.5193	11.1000	0.5727	−53.457
2048	FR	8	0.5129	11.1264	0.5760	−63.085
2048	BL	8	0.5095	11.1263	0.5779	−68.398
2048	BR	8	0.5064	11.0512	0.5736	−67.228

## Appendix B. Provenance of Superseded Latency Batches

The superseded latency values were reproduced from four distinct 12-trial groups, each with four legs and three repetitions, under the historical Qwen3.5:9b setting with no added latency. Table A2 reports sample standard deviations (n−1 denominator). The different standard deviations are not explained by switching between sample and population conventions. The endpoint is the wall-clock interval from the injected fault to the application-observed preset loading, not pure LLM generation time. These historical observations are not pooled with the current scheduler study, whose model, task, fault and measurement semantics differ.

**Table A2:** Source audit of superseded values, not new trials or replacement main results. All 12 observations in each historical batch are retained.

Historical Batch	*n*	Mean (s)	Sample SD (s)
Primary comparison	12	2.648500	1.642633
Ablation	12	2.239333	0.055241
Cross-model subset	12	2.223833	0.058353
Latency-sweep subset	12	2.195917	0.059332

The 7.858 s BR observation at historical trial timestamp 20260808_013131 contributes 91.436% of its batch’s total squared deviations from the mean. It is retained, not classified as an error or excluded. A unique emergency-log candidate matches its time window and BR scenario: fault to re-request is 0.101206 s, re-request to the completion-log timestamp is 2.123514 s, and completion-log timestamp to observed preset load is 5.633154 s. The worker’s recorded elapsed generation interval is 2.124 s. The logs lack shared trial/request identifiers, so the association is based on timing and scenario rather than an identity join.

This evidence localizes the large residual interval after the timestamp used for the completion log; it does not identify whether logging, output, queue insertion, thread scheduling, application observation or a wall-clock anomaly caused it. Calling the full 7.757 s re-request-to-load interval GPU inference time would be incorrect. Historical per-trial model digests, source hashes, host load and random seeds were not recorded, so a server-internal explanation cannot be recovered. The audit used exact preserved export identifiers rather than the old analysis script’s default date filter, which points to an earlier day. These provenance findings clarify the discrepancy while preserving the original records and their limits.
